# Serum copper and obesity among healthy adults in the National Health and Nutrition Examination Survey

**DOI:** 10.1371/journal.pone.0300795

**Published:** 2024-06-26

**Authors:** Menglu Liu, Changchang Fang, Kaibo Mei, Jitao Ling, Wanying Fu, Xinrui Qi, Peng Yu, Zhiwei Yan, Liang Xu, Yujie Zhao, Xiaozhong Li, Xiao Liu

**Affiliations:** 1 Department of Cardiology, Seventh People’s Hospital of Zhengzhou, Henan Provincial Key Laboratory of Arrhythmia, Henan Key Laboratory of Cardiac Remodeling and Transplantation, Zhengzhou, China; 2 Department of Endocrinology Medicine, The Second Affiliated Hospital of Nanchang University, Nanchang, Jiangxi, China; 3 Department of Anesthesiology, The People’s Hospital of Shangrao, Shangrao, Jiangxi, China; 4 Institute for the Study of Endocrinology and Metabolism of Jiangxi, Nanchang, Jiangxi, China; 5 College of Kinesiology, Shenyang Sport University, Shenyang, Liaoning, China; 6 Department of Cardiology, The Second Affiliated Hospital of Nanchang University, Nanchang, Jiangxi, China; 7 Department of Cardiology, Sun Yat-sen Memorial Hospital of Sun Yat-sen University, Guangzhou, Guangdong, China; 8 Guangzhou Key Laboratory of Molecular Mechanism and Translation in Major Cardiovascular Disease, Guangdong Provincial Key Laboratory of Malignant Tumor Epigenetics and Gene Regulation, Guangdong-Hong Kong Joint Laboratory for RNA Medicine, Guangzhou, Guangdong, China; Tehran University of Medical Sciences, ISLAMIC REPUBLIC OF IRAN

## Abstract

**Background:**

Copper (Cu) homeostasis are important processes in the cause of metabolic diseases, but the association between Cu and obesity remains unclear.

**Methods:**

Participants were drawn from the 2011–2016 National Health and Nutrition Examination Survey (NHANES). Weighted logistic regression assessed the associations of serum Cu concentrations (tertiles) with obesity and central obesity in individuals without comorbidities. Obesity was defined as a BMI ≥30.0 kg/m^2^, and central obesity was defined as a waist circumference ≥80 cm for women and ≥95 cm for men.

**Results:**

This cross-sectional study included 1,665 adults without comorbidities, representing 24,744,034 people (mean age 35.1 years, 48.5% female). High serum Cu levels (tertile 3: ≥19.19 μmol/L) were associated with higher odds of obesity (adjusted odds ratio [OR]: 4.48, 95% CI[confidence interval]: 2.44–8.32) and central obesity (OR: 2.36, 95% CI: 1.19–4.66) compared to low serum Cu levels (tertile 1: ≤15.64 μmol/L). The dose-response curve showed a nonlinear association between Cu levels and obesity (P-nonlinear = 0.02) and a linear association with central obesity (P-nonlinear = 0.21).

**Conclusion:**

This study suggests that higher serum Cu levels are associated with increased odds of obesity in healthy American adults.

## Introduction

Obesity is a well-known risk factor for the progression of diverse chronic diseases [1, 2]. The global odd of adults with overweight was 39% in 2016, 13% of whom were classified as individuals with obesity. The prevalence of obesity continues to increase, particularly in developed countries. In 2013–2014, the prevalence of obesity among American adults was 38% [3], and it is projected that by 2030, a majority of Americans will develop either obesity or overweight, with nearly 50% being classified as individuals with obesity [4].

The pathogenesis of obesity is complex and includes genetic, environmental, and nutritional factors [5]. Obesity and inflammation have been extensively linked, with obesity considered a state of chronic inflammation [6], elevated levels of oxidative stress and insulin resistance [7]. Cu generally exists in the environment and in food and is a vital trace element for humans [8]. Copper homeostasis is an emerging important process mediating the pathogenesis and progression of diverse diseases. Recent studies and our review suggest that copper homeostasis is closely related to the inflammatory response, oxidative stress, mitochondrial function [9], and inflammation [10, 11], which are shared mechanisms with obesity. Moreover, evidence from epidemiological studies has shown that serum Cu levels are greater with obesity than in adults without obesity [12–15]. However, the association between serum Cu concentration and the risk of obesity remains unclear. Our study aimed to assess this association in relatively healthy adults by focusing on participants without any comorbidities.

## Materials and methods

The present study was reported according to the Strengthening the Reporting of Observational Studies in Epidemiology (STROBE) Statement.

### Data source and study subjects

The National Health and Nutrition Examination Survey (NHANES) is a comprehensive cross-sectional study. The survey employs a sophisticated, stratified, and multistage probability sampling design. Since 1999, NHANES data have been released in 2-year cycles using this stratified multistage probability cluster design [16]. The research protocol received approval from the Research Ethics Review Board of the National Center for Health Statistics, and NHANES ensured that written informed consent was obtained from all participants. It is important to note that our analysis exclusively utilized publicly available data, ensuring the absence of personally identifiable information. The interview questionnaires and examination response rates are accessible to the public.

The NHANES 2011–2016 cycles included specific tests for serum Cu levels. The following inclusion criteria were applied to select participants: 1) aged ≥ 19 years old; 2) no missing Cu or body mass index (BMI) data; and 3) healthy status, defined as those without hypertension, diabetes, dyslipidemia, asthma, anemia, arthritis, heart failure, gout, coronary heart disease, myocardial infarction, angina pectoris, stroke, thyroid disease, chronic bronchitis, chronic obstructive pulmonary disease, liver disease, or cancer.

### Measurement

In the NHANES, participant information was obtained through interviews, anthropometric examinations, and blood sample tests. Blood pressure (BP) was measured by trained staff using a mercury sphygmomanometer after participants had rested for at least five minutes. Three measurements were averaged to obtain the final BP (in mmHg). Cigarette smoking status was determined based on responses to questions about lifetime smoking of at least 100 cigarettes and current smoking habits. Drinking status was determined using questionnaires that assessed whether participants had consumed at least 12 drinks in the past year or in their lifetime.

Physical activity (PA) was defined based on questionnaires assessing participation in vigorous or moderate recreational activities and daily sitting time. Blood samples were collected from participants at the mobile examination center and stored at -20°C before being sent to central laboratories. Standard methods were used to measure total cholesterol (TC), high-density lipoprotein cholesterol (HDL-C), hemoglobin A1c (HbA1c), alanine aminotransferase (ALT), uric acid (UA), and creatinine (CR) levels. Fasting triglyceride (TG), LDL cholesterol (LDL-C), and glucose levels were measured in eight-hour fasting blood samples. The estimated glomerular filtration rate (eGFR) was calculated using the Chronic Kidney Disease Epidemiology Collaboration equation [17].

### Measurement of serum Cu

A plasma dynamic reaction cell mass spectrometer (ELAN^®^ 6100 DRCPlus or ELAN^®^ DRC II, PerkinElmer Norwalk, CT, www.perkinelmer.com) was used to measure the baseline serum Cu levels in participants in the NHANES study. Detailed information on the laboratory analyses may be obtained from the laboratory procedure manual [18, 19]. The determined value of 0.39 μmol/L represented the lower limit of detection (LLOD) for Cu. The serum Cu levels of all participants surpassed this lower limit.

### Definition of obesity and central obesity

Obesity was defined by a BMI > = 30.0 kg/m^2^ [20]. The formula for BMI was weight in kilograms (kg) divided by height in meters squared (m^2^). Central obesity was defined as a waist circumference of 80 cm for women and 95 cm for men. Shoes and any hair accessories, jewelry, buns, or braids were removed before using a stadiometer with a fixed vertical backboard and an adjustable headpiece to measure standing height. Participants wore standard examination gowns with only underpants beneath the gowns before weighing in kilograms using a digital weight scale. Participants were told to lift the gown above the waist and clip it in the front to prevent it from obstructing the measurements of waist circumference. The details of the quality assurance and quality control measures used are described in the Anthropometry Procedures Manual [18].

### Covariates

The following covariates were used: age, sex, race (Mexican American, Other Hispanic, Non-Hispanic White, Non-Hispanic Black, and Other Race), marital status (unmarried, married, other), education (primary school graduate or below, middle/high/special school, college graduate or above), ratio of family income to poverty (PIR) (low: < 1.30, moderate: 1.31 to 3.50; high: ≥ 3.5), systolic BP, TyG index, TC, LDL-C, HDL-C, HbA1c, sedentary, moderate PA, current smoking, and drinking status.

### Statistical analysis

Continuous variables were presented as weighted means (standard errors), while categorical variables were presented as weighted frequency percentages. Serum Cu concentrations were analyzed through Z-standardization (mean = 0, SD = 1). Subjects were categorized into tertiles based on their serum Cu levels: Tertile 1 (<15.64 μmol/L), Tertile 2 (15.64–19.19 μmol/L), and Tertile 3 (≥19.19 μmol/L). Baseline differences across serum copper groups were examined using the chi-square test for categorical variables and the Kruskal–Wallis H test for continuous variables.

Pearson correlation coefficients were calculated to assess the correlations of serum Cu concentration with BMI and waist circumference. Weighted logistic regression was performed to evaluate associations of serum Cu concentrations with BMI and waist circumference, expressed as odds ratios (ORs) and 95% confidence intervals (95% CIs). Three models were constructed: crude; Model I, adjusted for age, sex, race, marital status, education, systolic BP, total cholesterol (TC), albumin, and uric acid (UA); and Model II, adjusted for Model I plus HbA1c, PIR, moderate PA, smoking status, and drinking status. Restricted cubic spline curves were used to evaluate the nonlinear association of Cu with obesity and central obesity.

Stratified analyses were conducted for various subgroups, including sex, age, systolic BP, HbA1c, TC, PIR, smoking status, drinking status, and moderate PA status. Subgroup analysis results were compared with those of the tertile 3 and tertile 1 groups. A multiple-imputation analysis was performed based on five replications, and the Markov-chain Monte Carlo method [21] and multiple imputation by chained equations was used to account for these covariates with missing data, including PIR, systolic BP, TyG index and drinking status. R software (version 4.1.3, www.R-project.org) was used to analyze the data. A two-sided p value of < 0.05 was regarded as statistically significant in all analyses.

## Results

### Baseline characteristics

**Fig 1:**
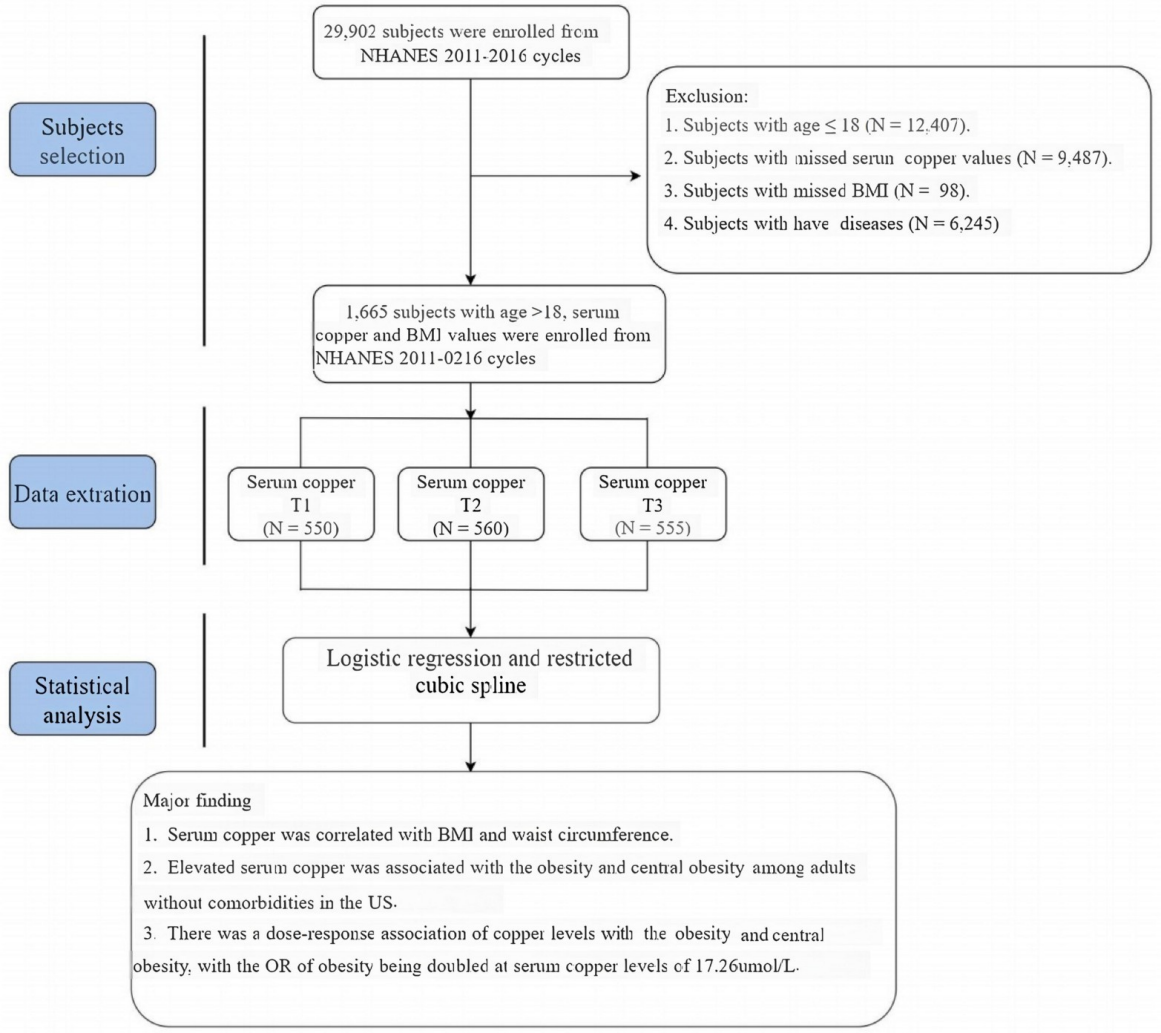
Study selection from the NHANES 2011–2016, workflow and major findings of this study. Abbreviations: NHANES: National Health and Nutrition Examination Survey.

The participant selection workflow is illustrated in Fig 1. We excluded participants aged <19 years, those with missing information on BMI and fasting serum Cu concentrations, and individuals with comorbidities; as a result, a total of 1,665 participants (mean age 35.1, SE ± 0.5; 48.5% female) were included in the data analysis. Table 1 displays the characteristics of the included participants, revealing a mean serum Cu level of 18.3 μmol/L (SE ± 0.3), a mean BMI of 27.4 kg/m^2^ (SE ± 0.3), and a mean waist circumference of 99.4 cm (SE ± 0.8). The majority of participants were non-Hispanic white (35.3%). A sensitivity analysis was also conducted to compare the baseline characteristics of the excluded and included individuals (S1 Table). Those without comorbidities were generally younger; more likely to be male; had a higher socioeconomic status; engaged in physical activity; had better renal function and lower BMI, waist circumference, and systolic blood pressure (SBP); were non-Hispanic white and married; and had lower levels of TC, TG, LDL-C, TyG index, fasting glucose, UA, serum Cu, and albumin (P < 0.01). A comparison of participants in tertile 3 (≥ 19.19 μmol/L) of serum Cu levels to those in tertile 1 (≤ 15.64 μmol/L) revealed that the former group exhibited higher rates of current smoking and drinking; better economic and educational status; a greater proportion of married individuals; and higher levels of serum TC, LDL-C, HbA1c, and eGFR (S2 Table).

**Table 1:** Weighted baseline characteristics by the tertile of the serum copper in adult Americans without comorbidities, Nation Health and Nutrition Examination Survey 2011–2016.

Characteristics	Population estimates	Observations
Copper, μmol/	18.3 (0.3)	1665
Age, year	35.1 (0.5)	1665
Female, % (n)	24,744,034 (48.5%)	807
BMI, kg/m^2^	27.4 (0.3)	< 0.01
Waist circumference, cm	94.4 (0.8)	1615
DBP, mm Hg	68.7 (0.6)	1530
SBP, mm Hg	116.1 (0.6)	1530
**Smoke status, % (n)**		
Never smoke	24,744,034 (66.5%)	1,107
Former smoke	24,744,034 (13.4%)	223
Current smoking	24,744,034 (20.1%)	335
**drinking, % (n)**		
Never drink	22,428,293 (17.8%)	296
Former drink	22,428,293 (9.7%)	162
Current drinking	22,428,293 (72.5%)	1207
**Race, % (n)**		
Mexican American	24,744,034 (15.7%)	261
Other Hispanic	24,744,034 (11.7%)	195
Non-Hispanic White	24,744,034 (35.3%)	588
Non-Hispanic Black	24,744,034 (17.4%)	290
Other Race	24,744,034 (19.9%)	331
**Marital status, % (n)**		
Never married	23,451,405 (34.5%)	574
Married	23451,405 (45.7%)	761
Other	23,451,405 (19.8%)	330
**Education status, % (n)**		
Primary school graduate or below	23,451,405 (5.1%)	85
Middle/high/special school	23,451,405 (35.1%)	584
College graduate or above	23,451,405 (59.8%)	99
**PIR, % (n)**		
low	23,120,568 (28.5%)	475
Moderate	23,120,568 (41.2%)	686
high	23,120568 (30.3%)	504
**Physical activity**		
Sedentary/min	377.4 (11.0)	
Moderate, % (n)	48.3%	804
Vigorous, % (n)	35.3%	588
**Laboratory results**		
TC, mmol/L	4.7 (0)	1,648
TG, mg/dL	1.2 (0)	7,34
HDL-C, mmol/L	1.4 (0)	1,648
LDL-C, mg/dL	2.8 (0)	734
Fasting glucose, mg/dL	5.4 (0)	762
eGFR, ml/min/1.73m2	133.9 (2.5)	1,645
UA, umol/L	304.5 (3.9)	1,650
TyG index	8.3 (0)	734
HbA1c, %	5.3 (0)	1,663
ALT, U/L	24.2 (0.8)	1,646
**Disease**		
Obesity, % (n)	24,744,034 (28.7%)	477
Central obesity, % (n)	24,744,034 (55.0%)	915

Note: Data are expressed as mean (SE) and numbers (percentage) as appropriate. All estimates were weighted to be nationally representative.

Abbreviations: PIR: Ratio of family income to poverty; BMI: body mass index; DBP: diastolic blood pressure; SBP: systolic blood pressure; HbA1c: glycated hemoglobin; TG: triglycerides; TC: total cholesterol; LDL-C: lower-density lipoprotein cholesterol; HDL-C: high-density lipoprotein cholesterol; ALT: Alanine Aminotransferase; TyG: triglycerides-glucose; Cr: creatinine; UA: uric acid; eGFR: estimated glomerular filtration rate.

### Serum Cu concentration, BMI and waist circumference

**Fig 2:**
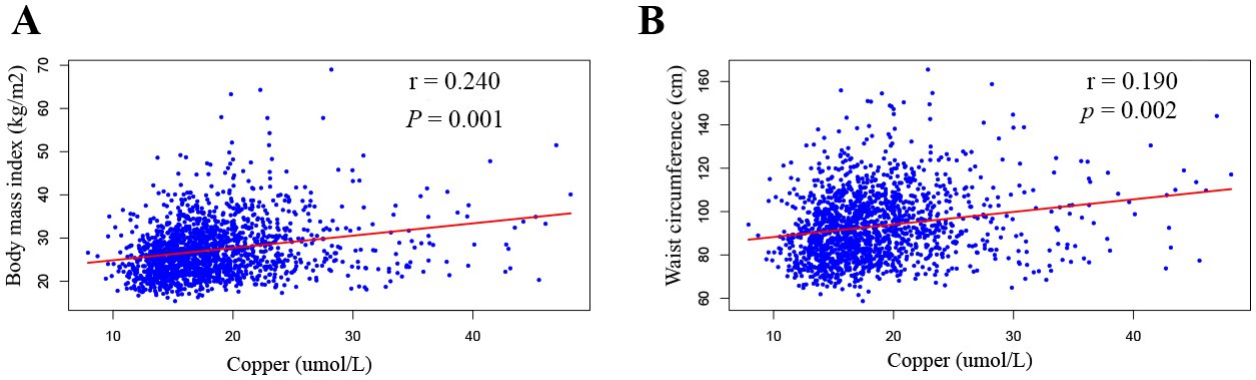
The correlation of serum Cu with body mass index and waist circumference. A: The correlation of serum Cu and body mass index. B: The correlation of the serum Cu waist circumference.

As shown in S1 Fig, BMI and waist circumference increased with tertiles of serum Cu levels (P < 0.05) in American adults without comorbidities. The serum Cu concentration was significantly correlated with BMI (r = 0.240; *P* = 0.001) and waist circumference (r = 0.190; *P* = 0.002) (Fig 2A and 2B).

S3 Table shows the associations between serum Cu concentrations and the prevalence of BMI and waist circumference in adults without comorbidities. A 1-SD increase in the serum Cu concentration was positively associated with BMI and waist circumference according to the crude and adjusted models (β > = 1.29).

### Examining the relationship between serum copper levels and obesity, particularly central obesity

As shown in Table 2, higher serum Cu concentrations were associated with an increased odds of obesity when assessed as a categorical variable. Compared to adults without comorbidities in tertile 1 (≤ 15.64 μmol/L), individuals in tertile 2 (15.64–19.19 μmol/L) and tertile 3 (≥ 19.19 μmol/L) had a greater risk of obesity according to the unadjusted model. These associations were consistent with the fully adjusted models (Tertile 2: aOR: 1.96, 95% CI: 1.03–3.72; Tertile 3: aOR: 4.48, 95% CI: 2.44–8.32). A positive association between the serum Cu concentration and the odds of obesity was detected when the serum Cu concentration was assessed as a continuous variable (per 1 SD increase; model II: aOR = 1.45, 95% CI = 1.16–1.82).

**Table 2:** Association of the serum copper with odds of obesity in adult Americans without comorbidities, Nation Health and Nutrition Examination Survey 2011–2016.

Copper, μmol/L	Case/N	Crude model OR (95%CI)	P	Model I OR (95%CI)	P	Model II OR (95%CI)	P
Per 1 SD increase	477/1665	1.30 (1.08,1.56)	0.008	1.46 (1.19,1.78)	< 0.001	1.45 (1.16,1.82)	0.004
Tertiles							
T1 (≤ 15.64)	93/550	Ref.	1.0	Ref.	1.0	Ref.	1.0
T2 (15.64–19.19)	175/560	2.23 (1.13,4.39)	0.025	2.42 (1.30,4.51)	0.009	1.96 (1.03,3.72)	0.052
T3 (≥ 19.19)	209/555	2.97 (1.62,5.45)	< 0.001	4.19 (2.32,7.54)	< 0.001	4.48 (2.44,8.23)	< 0.001
P for trend		< 0.0001		0.003		< 0.0001	

Note: Crude model was unadjusted for any factors; Model I was adjusted for age, gender, race, marital, education, SBP, TyG index, TC, ALT, and UA; Model II was adjusted for Model I, HbA1c, PIR, moderate PA, smoking status, drinking status.

Abbreviations: 95% CI: 95% confidence interval; OR: odds ratio; SBP: systolic blood pressure; TyG: triglyceride-glucose; TC: total cholesterol; UA: uric acid; HbA1c: glycated hemoglobin; ALT: alanine aminotransferase; PIR: Ratio of family income to poverty; PA: Physical activity.

Regarding central obesity, compared with Tertile 1, higher serum Cu levels (Tertile 2 and Tertile 3) were not significantly associated with the incidence of central obesity according to the crude model. S4 Table Unanimously, a higher serum Cu concentration (tertile 3) was associated with an increased odds of obesity according to the fully adjusted models (model II aOR: 2.36, 95% CI: 1.19–4.66). The analysis of continuous variables revealed a nonsignificant association between Cu concentration and the odds of central obesity according to the crude and adjusted models.

### Curve-fitting analysis the association of serum Cu concentration with both total and central obesity

**Fig 3:**
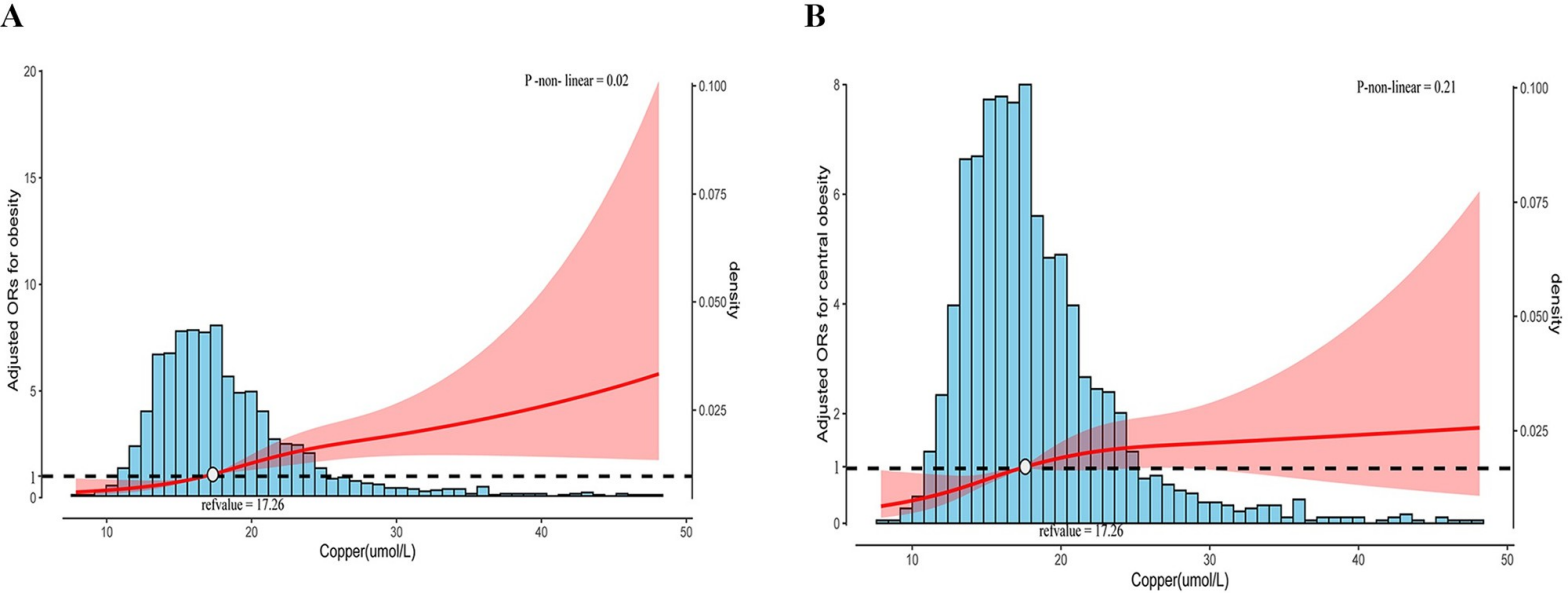
The odds ratio and the histogram of the probability distribution for obesity and central obesity. A: Odds ratio and histogram of the probability distribution for obesity; B: Odds ratio and histogram of the probability distribution for central obesity. A red curve with a light gray dotted line indicates an adjusted odds ratio with 95% CI for obesity based on serum Cu with reference to 17.26. The number of knots for the cubic spline curves was three in the model. Adjustment factors included Age, sex, race, marital status, education, SBP, TyG index, TC, albumin, UA, HbA1c, PIR, moderate PA, current smoking, and drinking status. Abbreviations: SBP: systolic blood pressure; TyG: triglyceride-glucose; TC: total cholesterol; UA: uric acid; HbA1c: glycated hemoglobin; PIR: ratio of family income to poverty; PA: physical activity.

The exposure–effect analysis unveiled a positive nonlinear association between serum Cu concentration and obesity (p-nonlinear = 0.02), and a positive linear association with central obesity (p-nonlinear = 0.21). At around 17.26 μmol/L of serum Cu concentration, the odds of both obesity and central obesity doubled (Fig 3A and 3B).

### Subgroup analyses and sensitivity analyses

**Fig 4:**
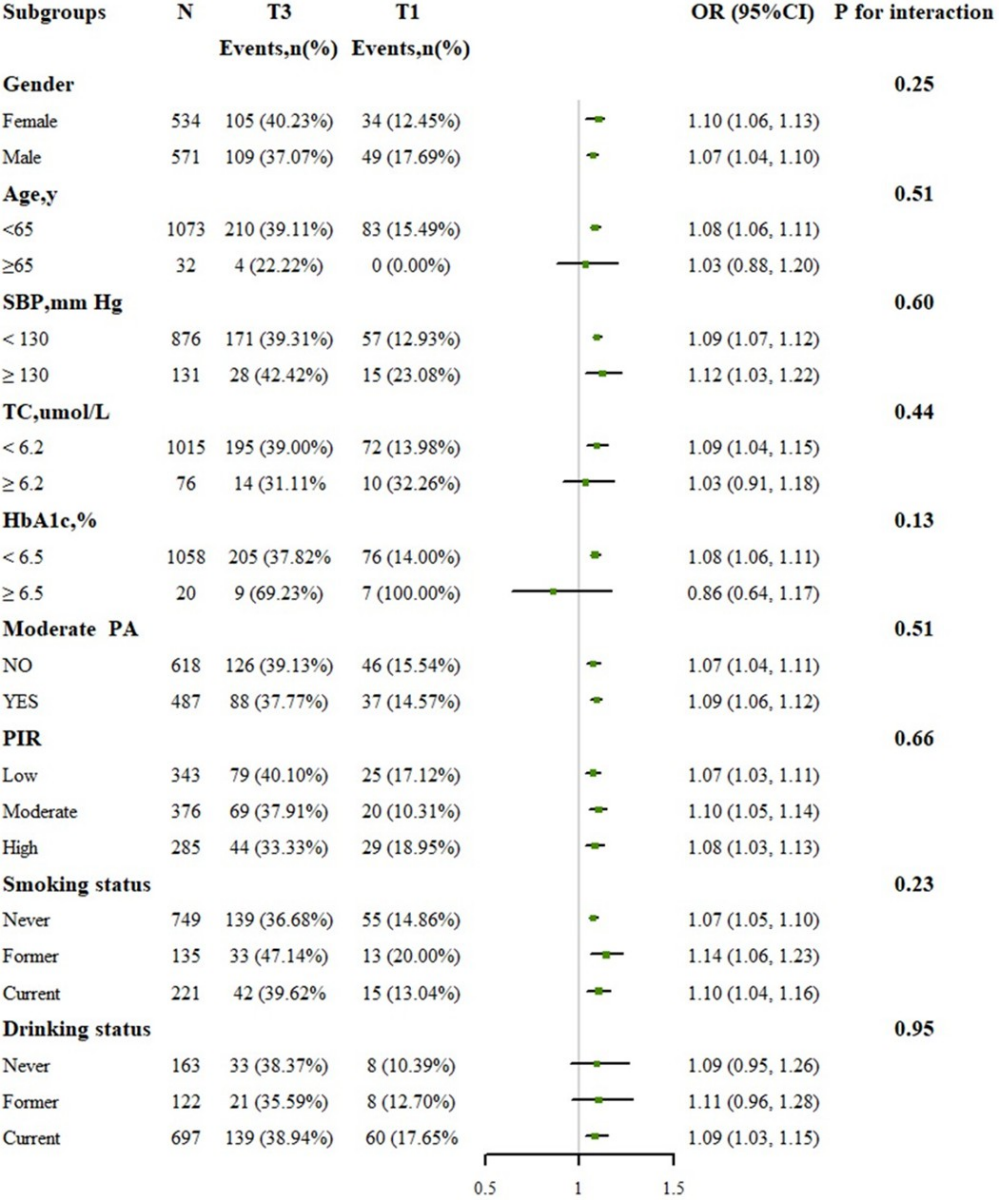
The association between serum Cu (T3 vs. T1) and obesity in various subgroups. The results were adjusted, if not stratified, for age, sex, race, marital status, education, SBP, TyG index, TC, ALT, UA, HbA1c, PIR, moderate PA, current smoking, and drinking status. Abbreviations: SBP: systolic blood pressure; TyG: triglyceride-glucose; TC: total cholesterol; ALT: alanine aminotransferase; UA: uric acid; HbA1c: glycated hemoglobin; PIR: ratio of family income to poverty; PA: physical activity.

Subgroup analyses, depicted in Fig 4, across various demographic and health-related factors including sex, age, marital status, SBP, HbA1c, TC, PIR, current smoking status, alcohol consumption, and moderate PA, did not alter the association between serum Cu concentrations and obesity (all p interactions >0.1). A sensitivity analysis using multiple imputation showed that missing variables did not impact the association between serum Cu levels and obesity (S5 and S6 Tables), consistent with the main findings from the complete case dataset (S7 Table). Furthermore, adjusting for serum zinc (Zn) to account for its potential influence confirmed the association between serum Cu and the incidence of obesity and central obesity (S8 Table).

## Discussion

### Major findings

On the basis of a national representative study of healthy participants, which represented 24,744,034 people, we showed that serum Cu concentrations were moderately correlated with BMI and waist circumference. Moreover, after we adjusted for demographic variables, lifestyle factors, and laboratory parameters, serum Cu levels were shown to be associated with a higher odd of obesity in healthy participants. These results were robust in the sensitivity analysis. Exposure–effect analysis revealed that the serum Cu concentration was approximately 17.26 μmol/L, at which point the odds of obesity doubled.

Obesity is prevalent in the US, necessitating effective, sustainable, and culturally tailored interventions due to vast disparities across groups and regions [4]. Elevated serum Cu levels have been linked to increased odds of overweight/total obesity and central obesity in US children and adolescents [22]. However, the association between serum Cu concentration and obesity in healthy adults remains unclear. This study represents the first attempt to evaluate Cu concentrations and their association with obesity odds in healthy adults.

### Comparisons with previous studies

At present, the association between serum Cu concentrations and obesity is unclear. For instance, a European study found no statistically significant difference in serum Cu levels between patients with morbid obesity and females without obesity [23], and a study in Asia showed that females with obesity have higher levels of serum Cu than healthy females [14]. A meta-analysis that included 21 articles with 2,299 subjects revealed that the serum Cu concentration was greater in children with obesity (standardized mean difference (SMD): 0.747; 95% CI: 0.16–1.32) and adults (SMD: 0.39; 95% CI: 0.02–0.76). This meta-analysis included only 6 studies of adults with obesity, and the sample sizes were limited. Furthermore, the meta-analysis included participants with normal and diseased conditions. Thus, the association between serum Cu levels and obesity in healthy adults has not been determined [12]. A study examining the association between serum Cu levels and obesity revealed that participants in the highest quintile of serum Cu levels had a greater risk of obesity (OR = 5.46, 95% CI = 3.31–8.98) and central obesity (OR = 5.64, 95% CI = 3.31–9.58) than did those in the lowest quintile [22]. Our study first focused on the association between serum Cu concentrations and obesity in adults, and we suggest that higher serum Cu concentrations are significantly associated with obesity and central obesity in healthy adults in the U.S.

There is emerging evidence showing that serum Cu increases the risk of metabolic syndrome [24], such as central obesity, insulin resistance, hypertension, glucose intolerance, and dyslipidemia [25]. For example, a cross-sectional study based on 128 nondiabetic patients with overweight and obesity revealed a significant positive association between the intake of Cu and the risk of insulin resistance [26]. Another cross-sectional study including 2,419 individuals who were normal weight but had metabolic obesity showed that those with copper levels between 79 and 131 μg/dl had no significant risk of metabolic syndrome (p = 0.46) compared to subjects with serum Cu levels less than 79 μg/dl. However, individuals with serum Cu levels above 131 μg/dl had a greater likelihood of metabolic syndrome (p = 0.01) after adjusting for age and sex [27]. Considering the strong association between obesity and metabolic syndrome, our study reinforced the above findings.

### Potential mechanism

Although the association between the serum Cu concentration and the risk of obesity remains to be determined, we hypothesized several potential mechanisms. The mechanism of obesity has been linked to oxidative stress, insulin resistance and inflammation [28–30]. Cu plays multiple roles in the human body, including as an integral element in various enzymes, such as Cu/zinc superoxide dismutase (Cu/Zn SOD). Thus, the overexpression of Cu/Zn SOD may contribute to oxidative stress [31] and the production of reactive oxygen species (ROS) [32].

Moreover, approximately 90% of the Cu in blood plasma is present in the form of ceruloplasmin [33]. Elevated serum ceruloplasmin levels have been linked to obesity due to increased pro-oxidant activity [33, 34]. Additionally, in patients with obesity, there is a notable increase in the levels of the copper-dependent enzyme semicarbazide-sensitive amine oxidase (SSAO) [35]. SSAO, which is a significant enzyme involved in oxidative deamination processes within the liver. It also plays intricate roles in inflammatory responses, glucose uptake stimulation, lipogenesis promotion, and contributing to weight gain [36, 37].

### Clinical implications

Previous studies have shown that higher levels of serum Cu levels increase the risk of metabolic abnormalities and are associated with a condition known as normal weight but are associated with metabolic obesity [27, 38]. Furthermore, there is a growing trend toward increased Cu intake as a dietary nutrient in U.S. adults [39]. However, the extent to which dietary Cu consumption prevents obesity remains unknown, and there are currently no clinical guidelines on this topic. Long-term randomized trials, although effective at reducing residual confounding, are challenging to implement practically, especially with regard to Cu exposure.

### Strength and limitations

Our study has several strengths. First, in this study, subjects from a national sample and those who had any comorbidities were excluded. Thus, we investigated the association between serum Cu concentration and obesity in relatively healthy subjects. Second, the positive associations are consistent in subgroup analyses by various covariates, which corresponds to consistency. Third, the dose–response analysis showed that individuals with higher serum Cu levels had a greater risk of total obesity and central obesity.

This study has several limitations. First, the cross-sectional design does not allow for establishing causality. Second, we should consider the possibility of residual confounding due to measurement errors in assessing confounding factors or unmeasured confounding factors. However, the similar results observed in sensitivity analyses indicate that the influence of residual confounding from measurement error is minimal. Third, the measures of body fat are diverse, and relying solely on BMI and waist circumference may not provide a comprehensive assessment of adiposity.

## Conclusions

The present study suggested that serum Cu levels are associated with obesity among healthy U.S. adults. However, additional prospective research is needed to determine this causal relationship.

## Supporting information

S1 Graphical abstract The abstract of the whole paper. (PDF)

S1 Table Weighted baseline characteristics by the tertile of the copper of adult Americans with comorbidities from the Nation Health and Nutrition Examination Survey 2011–2016. (DOCX)

S2 Table Weighted baseline characteristics by the tertile of the copper of adult Americans without comorbidities from the National Health and Nutrition Examination Survey 2011–2016. (DOCX)

S3 Table Adjusted regression coefficient (β) and 95% Confidence Intervals (95% CI) in BMI and waist circumference and serum copper in adult Americans without comorbidities from the Nation Health and Nutrition Examination Survey 2011–2016. (DOCX)

S4 Table Association of the copper with risk of central obesity in adult Americans without comorbidities from the Nation Health and Nutrition Examination Survey 2011–2016. (DOCX)

S5 Table Sensitive analysis of the association between Cu and risk of obesity in adult Americans based on the multiple-imputation analysis. (DOCX)

S6 Table The association between Cu and obesity in adult Americans based on the multiple-imputation of the MICE method. (DOCX)

S7 Table Association of the copper with risk of obesity in adult Americans without comorbidities in missing data from the Nation Health and Nutrition Examination Survey 2011–2016, complete case dataset. (DOCX)

S8 Table Association of the copper with risk of obesity in adult Americans without comorbidities after adjusted serum zinc from the Nation Health and Nutrition Examination Survey 2011–2016. (DOCX)

S1 Fig The body mass index and waist circumference values of tertiles of serum Cu. A: The body mass index and waist circumference values of tertiles of serum Cu; B: The waist circumference values of tertiles of serum Cu.
(DOCX)

S1 Checklist STROBE statement—Checklist of items that should be included in reports of cross-sectional studies. (DOC)
