# Supplementary material for: Serum copper and obesity among healthy adults in the National Health and Nutrition Examination Survey
Source: PLoS One. 2024 Jun 26;19(6):e0300795. doi: 10.1371/journal.pone.0300795 (PMC11206840; doi:10.1371/journal.pone.0300795)
Supplement: S1 Graphical abstract — (PDF) [file pone.0300795.s001.pdf]

GRAPHICAL ABSTRACT

Serum copper and obesity among healthy adults in the National Health and Nutrition Examination Survey

Background

Copper (Cu) is an important mineral in pathogenesis of metabolic disease. However, the association between Cu and obesity in healthy population is unclear.

Study subjects

1,665 adults without comorbidities

Sex: 48.5% female

Mean age: 35.1

Date source

National Health and Nutrition Examination Surveys (NHANES) 2011-2016

Obesity,n(%) 477(28.7)

Central obesity,n(%) 915(55.0%)

Results

1 Serum Cu and BMI and waist circumference

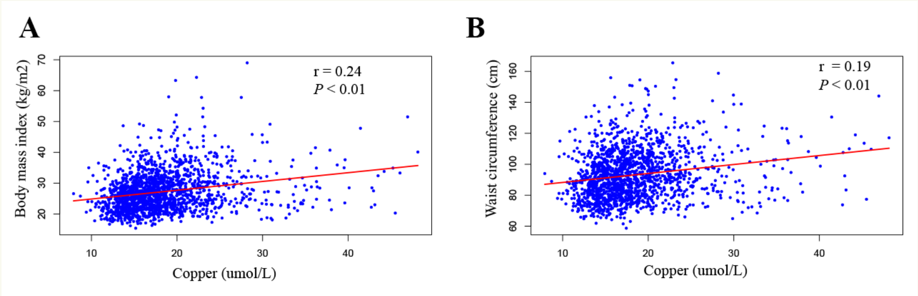

2 Curve-fitting association of Cu with total obesity and central obesity

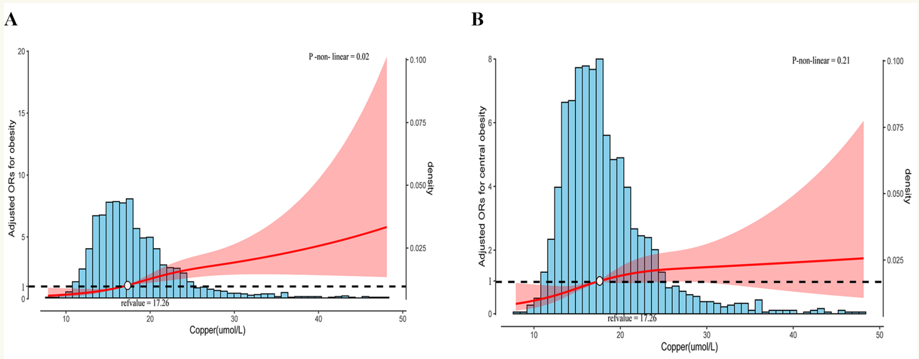

3 Association between Cu and obesity

a. Cu and total obesity

Copper, μmol/L	OR (95%CI)
T1 (≤ 15.64)	Ref.
T2 (15.64- 19.19)	1.95 (1.02,3.70)
T3 (≥ 19.19)	4.44 (2.42,8.14)

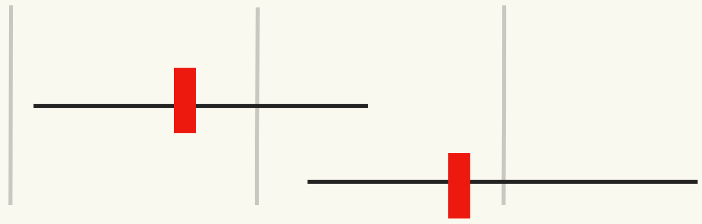

b. Cu and central obesity

Copper, μmol/L	OR (95%CI)
T1 (≤ 15.64)	Ref.
T2 (15.64- 19.19)	1.80 (0.98,3.30)
T3 (≥ 19.19)	2.37 (1.21,4.62)

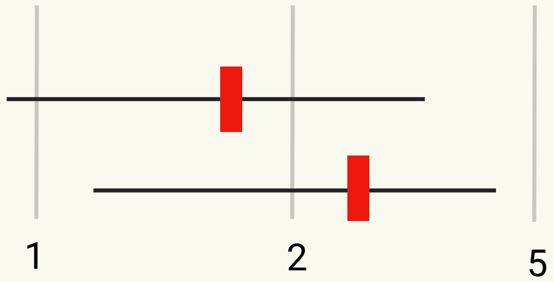

Conclusion

Present study suggest excessive serum copper levels is associated with a higher prevalence of obesity in healthy America adults.
