# Supplementary material for: Serum copper and obesity among healthy adults in the National Health and Nutrition Examination Survey
Source: PLoS One. 2024 Jun 26;19(6):e0300795. doi: 10.1371/journal.pone.0300795 (PMC11206840; doi:10.1371/journal.pone.0300795)
Supplement: S1 Table — (DOCX) [file pone.0300795.s002.docx]

**Table S1 Weighted baseline characteristics by the Tertile of the Copper of adult Americans with comorbidities from the Nation Health and Nutrition Examination Survey 2011-2016**

| **Characteristics** | **Comorbidities** | **Healthy** | **P** |
| --- | --- | --- | --- |
| Total, % (n) | 68.1 (3552) | 31.9 (1665) | < 0.01 |
| Copper, μmol/L | 18.7 (0.2) | 18.3 (0.3) | 0.20 |
| Age, year | 52.6 (0.5) | 35.1 (0.5) | < 0.01 |
| Female, % (n) | 50.2 (1783) | 48.5 (807) | < 0.01 |
| BMI, kg/m2 | 29.8 (0.3) | 27.4 (0.3) | < 0.01 |
| Waist circumference, cm | 102.4 (0.6) | 94.4 (0.8) | < 0.01 |
| DBP, mm Hg | 69 (0.5) | 68.7 (0.6) | 0.39 |
| SBP, mm Hg | 124 (0.7) | 116.1 (0.6) | < 0.01 |
| **Smoke status, % (n)** |  |  | 0.78 |
| Never smoke | 51.2 (1818) | 66.5 (1107) |  |
| Former smoke | 29.4 (1045) | 13.4 (223) |  |
| Current smoking | 19.4 (689) | 20.1 (335) |  |
| **drinking, % (n)** |  |  | 0.06 |
| Never drink | 12.0 (426) | 17.8 (296) |  |
| Former drink | 10.2 (362) | 9.7 (162) |  |
| Current drinking | 77.8 (2764) | 72.5 (1207) |  |
| **Race % (n)** |  |  | < 0.01 |
| Mexican American | 6.6 (234) | 15.7 (261) |  |
| Other Hispanic | 6.0 (213) | 11.7 (195) |  |
| Non-Hispanic White | 69.1 (2454) | 35.3 (588) |  |
| Non-Hispanic Black | 11.3 (401) | 17.4 (290) |  |
| Other Race | 7.0 (249) | 19.9 (331) |  |
| **Marital status, % (n)** |  |  | 0.02 |
| Never married | 14.4 (511) | 34.5 (574) |  |
| Married | 58.5 (2078) | 45.7 (761) |  |
| Other | 27.1 (963) | 19.8 (330) |  |
| **Education status, % (n)** |  |  | 0.43 |
| Primary school graduate or below | 6.4 (227) | 5.1 (85) |  |
| Middle/high/special school | 31.9 (1133) | 35.1 (584) |  |
| College graduate or above | 61.7 (2192) | 59.8 (996) |  |
| **PIR, % (n)** |  |  | < 0.01 |
| low | 22.5 (799) | 28.5 (475) |  |
| Moderate | 37.0 (1314) | 41.2 (686) |  |
| high | 40.5 (1439) | 30.3 (504) | 0.04 |
| **Physical activity** |  |  | < 0.01 |
| Sedentary/min | 417.2 (7.8) | 377.4 (11.0) |  |
| Moderate, % (n) | 41.2 (1463) | 48.3 (804) |  |
| Vigorous, % (n) | 21.3 (757) | 35.3 (588) |  |
| **Laboratory results** |  |  |  |
| TC, mmol/L | 5.1 (0) | 4.7 (0) | < 0.01 |
| TG, mmol/L | 1.4 (0) | 1.2 (0) | < 0.01 |
| HDL-C, mmol/L | 1.4 (0) | 1.4 (0) | 0.66 |
| LDL-C, mmol/L | 3.0 (0) | 2.8 (0) | < 0.01 |
| Fasting glucose, mmol/L | 6.1 (0) | 5.4 (0) | < 0.01 |
| eGFR ml/min/1.73m2 | 116.5 (1.6) | 133.9 (2.5) | < 0.01 |
| UA, umol/L | 328.4 (3.1) | 304.5 (3.9) | < 0.01 |
| TyG index | 8.6 (0) | 8.3 (0) | < 0.01 |
| HbA1c, % | 5.8 (0) | 5.3 (0) | < 0.01 |
| Albumin, g/L | 26.8 (0.6) | 24.2 (0.8) | < 0.01 |
| **Disease** |  |  |  |
| Obesity, % (n) | 40.5 (1439) | 28.7 (477) | < 0.01 |
| Central obesity, % (n) | 79.3 (2817) | 55.0 (915) | < 0.01 |

Note: Data are expressed as mean (SE) and numbers (percentage) as appropriate. All estimates were weighted to be nationally representative.

Abbreviations: PIR: Ratio of family income to poverty; BMI: body mass index; DBP: diastolic blood pressure; SBP: systolic blood pressure; HbA1c: glycated hemoglobin; TG: triglycerides; TC: total cholesterol; LDL-C: lower-density lipoprotein cholesterol; HDL-C: high-density lipoprotein cholesterol; ALT: Alanine Aminotransferase; TyG: triglycerides-glucose; Cr: creatinine; UA: uric acid; eGFR: estimated glomerular filtration rate.
