# Supplementary material for: Serum copper and obesity among healthy adults in the National Health and Nutrition Examination Survey
Source: PLoS One. 2024 Jun 26;19(6):e0300795. doi: 10.1371/journal.pone.0300795 (PMC11206840; doi:10.1371/journal.pone.0300795)
Supplement: S2 Table — (DOCX) [file pone.0300795.s003.docx]

**Table S****2 Weighted baseline characteristics by the tertile of the copper of adult Americans** **without comorbidities from the national health and Nutrition Examination Survey 2011-2016**

| **Characteristics** | **Tertiles of serum Cu** | | | **P** |
| --- | --- | --- | --- | --- |
|  | T1 (≤ 15.64μmol/L)  N = 550 | T2 (15.64-19.19μmol/L)  N = 560 | T3 (≥ 19.19μmol/L)  N = 555 |  |
| Copper, μmol/ | 14.7 (0.1) | 22.0 (0.4) | 24.1 (0.4) | < 0.01 |
| Age, year | 34.6 (1.2) | 36.3 (0.9) | 34.2 (1.0) | 0.79 |
| Female, % (n) | 52.3 (287) | 46.7 (266) | 45.9 (254) | 0.29 |
| BMI, kg/m^2^ | 25.5 (0.4) | 27.5 (0.6) | 29.7 (0.7) | < 0.01 |
| Waist circumference, cm | 90.0 (1.3) | 94.7 (1.5) | 98.5 (1.6) | < 0.01 |
| DBP, mm Hg | 70.3 (0.9) | 68.6 (0.8) | 67.2 (1.0) | 0.02 |
| SBP, mm Hg | 116.9 (1.2) | 116.1 (1.6) | 115.2 (1.1) | 0.27 |
| **Smoke status, % (n)** |  |  |  | 0.88 |
| Never smoke | 66.0 (363) | 69.4 (389) | 63.9 (355) |  |
| Former smoke | 14.1 (78) | 13.6 (76) | 12.4 (69) |  |
| Current smoking | 19.9 (109) | 17.0 (95) | 23.7 (131) |  |
| **drinking, % (n)** |  |  |  | 0.03 |
| Never drink | 22.7 (125) | 18.7 (105) | 11.9 (66) |  |
| Former drink | 9.8 (54) | 9.1 (51) | 10.3 (57) |  |
| Current drinking | 67.4 (371) | 72.2 (404) | 77.8 (432) |  |
| **Race, % (n)** |  |  |  | 0.41 |
| Mexican American | 13.2 (73) | 19.9 (111) | 13.8 (77) |  |
| Other Hispanic | 10.3 (57) | 11.4 (64) | 13.5 (74) |  |
| Non-Hispanic White | 35.2 (194) | 34.9 (195) | 35.8 (199) |  |
| Non-Hispanic Black | 18.5 (102) | 13.4 (75) | 20.4 (113) |  |
| Other Race | 22.8 (124) | 20.4 (115) | 16.5 (92) |  |
| **Marital status, % (n)** |  |  |  | 0.24 |
| Never married | 35.1 (193) | 34.8 (195) | 33.5 (186) |  |
| Married | 43.2 (238) | 47.6 (267) | 46.2 (256) |  |
| Other | 21.7 (119) | 17.6 (98) | 20.3 (113) |  |
| **Education status, % (n)** |  |  |  | 0.04 |
| Primary school graduate or below | 3.5 (20) | 6.0 (33) | 5.8 (32) |  |
| Middle/high/special school | 38.0 (209) | 32.5 (182) | 34.8 (193) |  |
| College graduate or above | 58.5 (321) | 61.5 (345) | 59.4 (330) |  |
| **PIR, % (n)** |  |  |  | < 0.01 |
| low | 24.3 (134) | 26.2 (146) | 35.2 (195) |  |
| Moderate | 38.0 (209) | 46.3 (260) | 39.1 (217) |  |
| high | 37.7 (207) | 27.5 (154) | 25.7 (143) |  |
| **Physical activity** |  |  |  | 0.43 |
| Sedentary/min | 407.6 (23.7) | 352.8(17.1) | 374.9 (17.3) |  |
| Moderate, % (n) | 52.0 (286) | 46.2 (258) | 47.0 (260) |  |
| Vigorous, % (n) | 43.9 (241) | 30.6 (171) | 32.0 (177) |  |
| **Laboratory results** |  |  |  |  |
| TC, mmol/L | 4.6 (0.1) | 4.7 (0.1) | 4.9 (0.1) | < 0.01 |
| TG, mg/dL | 1.1 (0.1) | 1.2 (0.1) | 1.2 (0) | 0.78 |
| HDL-C, mmol/L | 1.4 (0) | 1.4 (0) | 1.5 (0) | 0.73 |
| LDL-C, mg/dL | 2.7 (0.1) | 2.8 (0.1) | 2.9 (0.1) | 0.02 |
| Fasting glucose, mg/dL | 5.4 (0) | 5.5 (0.1) | 5.4 (0.1) | 0.98 |
| eGFR, ml/min/1.73m2 | 127.5 (3.5) | 131.9 (3.6) | 142.8 (5.4) | 0.03 |
| UA, umol/L | 303.4 (7.9) | 305.3 (7.2) | 304.6 (6.1) | 0.90 |
| TyG index | 8.3 (0.1) | 8.3 (0.1) | 8.4 (0) | 0.22 |
| HbA1c, % | 5.2 (0) | 5.4 (0) | 5.4 (0) | 0.01 |
| ALT, U/L | 25.1 (1.4) | 25.1 (1.2) | 22.3 (1.3) | 0.17 |
| **Disease** |  |  |  |  |
| Obesity, % (n) | 16.9 (93) | 31.2 (175) | 37.7 (209) | < 0.01 |
| Central obesity, % (n) | 49.4 (272) | 57.6 (322) | 57.8 (321) | < 0.01 |

Note: Data are expressed as mean (SE) and numbers (percentage) as appropriate. All estimates were weighted to be nationally representative.

Abbreviations: PIR: Ratio of family income to poverty; BMI: body mass index; DBP: diastolic blood pressure; SBP: systolic blood pressure; HbA1c: glycated hemoglobin; TG: triglycerides; TC: total cholesterol; LDL-C: lower-density lipoprotein cholesterol; HDL-C: high-density lipoprotein cholesterol; TyG: triglycerides-glucose; ALT: Alanine Aminotransferase; Cr: creatinine; UA: uric acid; eGFR: estimated glomerular filtration rate.
