# Supplementary material for: Serum copper and obesity among healthy adults in the National Health and Nutrition Examination Survey
Source: PLoS One. 2024 Jun 26;19(6):e0300795. doi: 10.1371/journal.pone.0300795 (PMC11206840; doi:10.1371/journal.pone.0300795)
Supplement: S3 Table — (DOCX) [file pone.0300795.s004.docx]

**TABLE S3 Adjusted regression** **coefficient (β) and 95% Confidence Intervals (95% CI) in BMI and waist circumference and serum copper in adult Americans without comorbidities from the Nation Health and Nutrition Examination Survey 2011-2016**

| Copper, mmol/L | Crude model  β (95%CI) | Adjusted R^2^ | Model I  β (95%CI) | Adjusted R^2^ | Model II  β (95%CI) | Adjusted R^2^ |
| --- | --- | --- | --- | --- | --- | --- |
| BMI |  |  |  |  |  |  |
| Per 1 SD increase | 1.29 (0.66,1.94) * | 0.05 | 1.44 (0.80,2.07) * | 0.24 | 1.41 (0.72,2.10) * | 0.27 |
| Waist circumference |  |  |  |  |  |  |
| Per 1 SD increase | 2.28 (0.69,3.88) * | 0.04 | 2.67 (1.26,4.08) * | 0.29 | 2.58 (1.11,4.07) * | 0.31 |

Note: Crude model was unadjusted for any factors; Model I was adjusted for age, gender, race, marital, education, SBP, TyG index, TC, ALT, and UA; Model II was adjusted for Model I, HbA1c, PIR, moderate PA, smoking status, drinking status. * P < 0.01

Abbreviations: 95% CI: 95% confidence interval; β: effect size; SBP: systolic blood pressure; TyG: triglyceride-glucose; TC: total cholesterol; UA: uric acid; HbA1c: glycated hemoglobin; ALT: alanine aminotransferase; PIR: Ratio of family income to poverty; PA: Physical activity.
