# Supplementary material for: Serum copper and obesity among healthy adults in the National Health and Nutrition Examination Survey
Source: PLoS One. 2024 Jun 26;19(6):e0300795. doi: 10.1371/journal.pone.0300795 (PMC11206840; doi:10.1371/journal.pone.0300795)
Supplement: S4 Table — (DOCX) [file pone.0300795.s005.docx]

**Table S4 Association of the copper with risk of** **central obesity in adult Americans without comorbidities from the Nation Health and Nutrition Examination Survey 2011-2016**

| Copper, μmol/L | Case/N | Crude model  OR (95%CI) | P | Model I  OR (95%CI) | P | Model II  OR (95%CI) | P |
| --- | --- | --- | --- | --- | --- | --- | --- |
| Per 1 SD increase | 915/1665 | 1.02 (0.86,1.22) | 0.803 | 1.12 (0.93,1.34) | 0.228 | 1.15 (0.92,1.43) | 0.224 |
| Tertiles |  |  |  |  |  |  |  |
| T1 (≤ 15.64) | 272/550 | Ref. | 1.0 | Ref. | 1.0 | Ref. | 1.0 |
| T2 (15.64- 19.19) | 322/560 | 1.40 (0.83,2.35) | 0.215 | 1.92 (1.13,3.25) | 0.022 | 1.78 (0.98,3.27) | 0.070 |
| T3 (≥ 19.19) | 321/555 | 1.41 (0.87,2.29) | 0.174 | 2.10 (1.15,3.86) | 0.023 | 2.36 (1.19,4.66) | 0.022 |
| P for trend |  | 0.26 |  | < 0.0001 |  | < 0.0001 |  |

Note: Crude model was unadjusted for any factors; Model I was adjusted for M age, gender, race, marital, education, SBP, TyG index, TC, ALT, and UA; Model II was adjusted for Model I, HbA1c, PIR, moderate PA, smoking status, drinking status.

Abbreviations: 95% CI: 95% confidence interval; OR: odds ratio; SBP: systolic blood pressure; TyG: triglyceride-glucose; TC: total cholesterol; UA: uric acid; HbA1c: glycated hemoglobin; PIR: Ratio of family income to poverty; PA: Physical activity.
