# Supplementary material for: Serum copper and obesity among healthy adults in the National Health and Nutrition Examination Survey
Source: PLoS One. 2024 Jun 26;19(6):e0300795. doi: 10.1371/journal.pone.0300795 (PMC11206840; doi:10.1371/journal.pone.0300795)
Supplement: S5 Table — (DOCX) [file pone.0300795.s006.docx]

**Table S5 Sensitive analysis of the association between Cu and risk of obesity in adult Americans based on the multiple-imputation analysis**

| Item | B | SE | OR (95%CI) | P |
| --- | --- | --- | --- | --- |
| 1 | 0.39 | 0.10 | 1.48 (1.20,1.81) | 0.001 |
| 2 | 0.39 | 0.10 | 1.48 (1.21,1.80) | 0.001 |
| 3 | 0.40 | 0.10 | 1.49 (1.21,1.82) | 0.001 |
| 4 | 0.39 | 0.10 | 1.48 (1.21,1.80) | 0.001 |
| 5 | 0.38 | 0.10 | 1.46 (1.20,1.78) | 0.001 |
| Pooled estimates | 0.39 | 0.10 | 1.48 (1.21,1.81) | 0.001 |

Note: Multiple-imputation analysis which is based on 5 replications and the Markov-chain Monte Carlo method in the SAS MI procedure

. The five items are replenished data from the Multiple-imputation procedure that used 5 replications and the Markov-chain Monte Carlo method based on PIR, SBP, TyG index, and drinking. The 5 replenished data were applied to investigate the association between Cu and obesity. Cu as a continuous variable was entered into the binary-weighted logistic regression analysis, which was used to evaluate the association between Cu and obesity. The result was expressed as odds ratios and 95% confidence intervals with the pre-defined model. after adjusting for age, gender, race, marital, education, SBP, TyG index, TC, ALT, UA, HbA1c, PIR, moderate PA, current smoking, drink status. Then, pooled estimates from five imputed data.

Abbreviations: NHANES: Nation Health and Nutrition Examination Survey; SBP: systolic blood pressure; TC: total cholesterol; HbA1c: glycated hemoglobin; TyG: triglyceride-glucose; ALT: Alanine Aminotransferase; UA: uric acid; PIR: Ratio of family income to poverty; PA: Physical activity.
