# Supplementary material for: Serum copper and obesity among healthy adults in the National Health and Nutrition Examination Survey
Source: PLoS One. 2024 Jun 26;19(6):e0300795. doi: 10.1371/journal.pone.0300795 (PMC11206840; doi:10.1371/journal.pone.0300795)
Supplement: S6 Table — (DOCX) [file pone.0300795.s007.docx]

**Table S6 The association between Cu and obesity in adult Americans based on the multiple-imputation of the MICE method.**

| Item | B | SE | OR (95%CI) | P |
| --- | --- | --- | --- | --- |
| 1 | 0.10 | 0.01 | 1.10 (1.07,1.12) | 0.001 |
| 2 | 0.10 | 0.01 | 1.10 (1.07,1.12) | 0.001 |
| 3 | 0.10 | 0.01 | 1.10 (1.07,1.12) | 0.001 |
| 4 | 0.09 | 0.01 | 1.09 (1.07,1.12) | 0.001 |
| 5 | 0.09 | 0.01 | 1.09 (1.07,1.12) | 0.001 |
| Pooled estimates | 0.09 | 0.01 | 1.09 (1.06,1.12) | 0.001 |
