# Supplementary material for: Serum copper and obesity among healthy adults in the National Health and Nutrition Examination Survey
Source: PLoS One. 2024 Jun 26;19(6):e0300795. doi: 10.1371/journal.pone.0300795 (PMC11206840; doi:10.1371/journal.pone.0300795)
Supplement: S7 Table — (DOCX) [file pone.0300795.s008.docx]

**TABLE S7 Association of the copper with risk of obesity in adult Americans without comorbidities in missing data from the Nation Health and Nutrition Examination Survey 2011-2016, complete case dataset**

| Copper, μmol/L | Case/N | Crude Model  OR (95%CI) | P | Model I  OR (95%CI) | P | Model II  OR (95%CI) | P |
| --- | --- | --- | --- | --- | --- | --- | --- |
| Total obesity |  |  |  |  |  |  |  |
| Per 1 unit increase | 272/1171 | 1.27 (1.02,1.58) | 0.037 | 1.47 (1.17,1.86) | 0.004 | 1.48 (1.18,1.87) | < 0.001 |
| Tertiles |  |  |  |  |  |  |  |
| T1 (≤ 15.764) | 57/387 | Ref. | 1.0 | Ref. | 1.0 | Ref. | 1.0 |
| T2 (15.64- 19.0) | 104/393 | 1.68 (0.76,3.72) | 0.202 | 1.76 (0.84,3.70) | 0.145 | 1.75 (0.89,3.44) | 0.120 |
| T3 (≥ 19.04) | 151/391 | 3.11 (1.51,6.38) | 0.003 | 4.56 (2.34,8.89) | < 0.001 | 4.49 (2.41,8.38) | < 0.001 |
| P for trend |  | < 0.0001 |  | < 0.0001 |  | 0.0254 |  |
| Central obesity |  |  |  |  |  |  |  |
| Per 1 unit increase | 482/1171 | 0.97 (0.78,1.20) | 0.771 | 1.16 (0.96,1.41) | 0.124 | 1.20 (0.99,1.46) | 0.068 |
| Tertiles |  |  |  |  |  |  |  |
| T1 (≤ 15.64) | 171/387 | Ref. | 1.0 | Ref. | 1.0 | Ref. | 1.0 |
| T2 (15.64- 19.04) | 201/393 | 1.21 (0.68,2.14) | 0.521 | 1.62 (0.90,2.90) | 0.136 | 1.65 (0.91,2.97) | 0.111 |
| T3 (≥ 19.04) | 223/391 | 1.32 (0.72,2.42) | 0.366 | 2.31 (1.21,4.43) | 0.017 | 2.49 (1.33,4.67) | 0.009 |
| P for trend |  | 0.050 |  | < 0.0001 |  | < 0.0001 |  |

Note: Crude model was unadjusted for any factors; Model I was adjusted for age, gender, race, marital, education, SBP, TC, ALT, and UA; Model II was adjusted for Model I, HbA1c, PIR, moderate PA, smoking status, drinking status.

### Abbreviations: 95% CI: 95% confidence interval; OR: odds ratio; SBP: systolic blood pressure; TC: total cholesterol; UA: uric acid; HbA1c: glycated hemoglobin; ALT: alanine aminotransferase; PIR: Ratio of family income to poverty; PA: Physical activity.
