# Supplementary material for: Serum copper and obesity among healthy adults in the National Health and Nutrition Examination Survey
Source: PLoS One. 2024 Jun 26;19(6):e0300795. doi: 10.1371/journal.pone.0300795 (PMC11206840; doi:10.1371/journal.pone.0300795)
Supplement: S8 Table — (DOCX) [file pone.0300795.s009.docx]

**TABLE S8**  **Association of the copper with risk of obesity in adult Americans without comorbidities after adjusted serum zinc from the Nation Health and Nutrition Examination Survey 2011-2016**

| Copper, μmol/L | Case/N | Model I  OR (95%CI) | P | Model II  OR (95%CI) | P |
| --- | --- | --- | --- | --- | --- |
| Total obesity |  |  |  |  |  |
| Per 1 unit increase | 477/1665 | 1.45 (1.16,1.82) | 0.004 | 1.45 (1.16,1.82) | 0.004 |
| Tertiles |  |  |  |  |  |
| T1 (≤ 15.64) | 93/550 | Ref. | 1.0 | Ref. | 1.0 |
| T2 (15.64- 19.19) | 175/560 | 1.96 (1.03,3.72) | 0.052 | 1.95 (1.02,3.70) | 0.055 |
| T3 (≥ 19.19) | 209/555 | 4.48 (2.44,8.23) | < 0.001 | 4.44 (2.42,8.14) | < 0.001 |
| P for trend |  | < 0.0001 |  | < 0.0001 |  |
| Central obesity |  |  |  |  |  |
| Per 1 unit increase | 915/1665 | 1.15 (0.92,1.43) | 0.224 | 1.15 (0.92,1.43) | 0.228 |
| Tertiles |  |  |  |  |  |
| T1 (≤ 15.64) | 272/550 | Ref. | 1.0 | Ref. | 1.0 |
| T2 (15.64- 19.19) | 322/560 | 1.78 (0.98,3.27) | 0.070 | 1.80 (0.98,3.30) | 0.072 |
| T3 (≥ 19.19) | 321/555 | 2.36 (1.19,4.66) | 0.022 | 2.37 (1.21,4.62) | 0.020 |
| P for trend |  | < 0.0001 |  | < 0.0001 |  |

Note: Model I was adjusted for age, gender, race, marital, education, SBP, TyG index, TC, ALT, UA, HbA1c, PIR, moderate PA, smoking status, and drinking status. Model II was adjusted for age, gender, race, marital, education, SBP, TyG index, TC, ALT, UA, HbA1c, PIR, moderate PA, smoking status, drinking status, and zinc.

### Abbreviations: 95% CI: 95% confidence interval; OR: odds ratio; SBP: systolic blood pressure; TyG: triglyceride-glucose; TC: total cholesterol; UA: uric acid; HbA1c: glycated hemoglobin; ALT: alanine aminotransferase; PIR: Ratio of family income to poverty; PA: Physical activity.
