# Supplementary material for: Serum copper and obesity among healthy adults in the National Health and Nutrition Examination Survey
Source: PLoS One. 2024 Jun 26;19(6):e0300795. doi: 10.1371/journal.pone.0300795 (PMC11206840; doi:10.1371/journal.pone.0300795)
Supplement: S1 Fig — A: The body mass index and waist circumference values of tertiles of serum Cu; B: The waist circumference values of tertiles of serum Cu. (DOCX) [file pone.0300795.s010.docx]

**Figure S1 The body mass index and waist circumference values of tertiles of serum Cu. A: The body mass index and waist circumference values of tertiles of serum Cu; B: The waist circumference values of tertiles of serum Cu.**


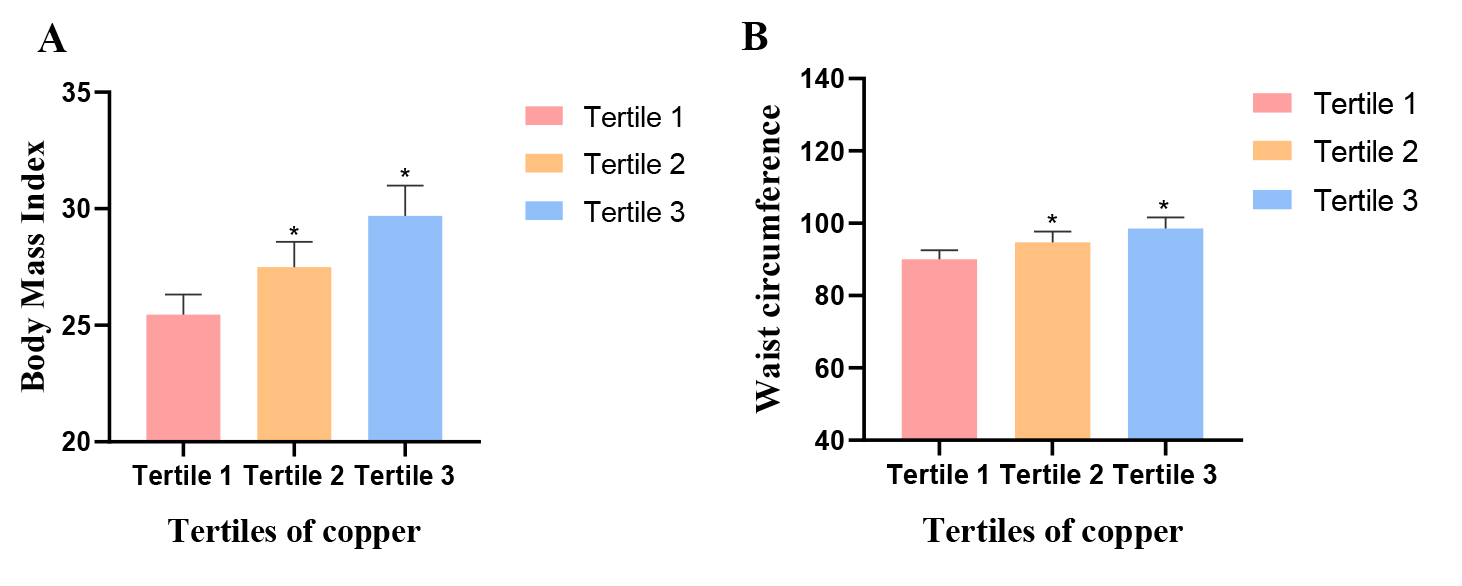


**Figure S1** The body mass index and waist circumference values of tertiles of serum Cu. A: The body mass index and waist circumference values of tertiles of serum Cu; B: The waist circumference values of tertiles of serum Cu.
